# Hyperoxia reduces insulin release and induces mitochondrial dysfunction with possible implications for hyperoxic treatment of neonates

**DOI:** 10.14814/phy2.13447

**Published:** 2017-10-16

**Authors:** Ingrid Hals, Tsuyoshi Ohki, Rinku Singh, Zuheng Ma, Anneli Björklund, Chandima Balasuriya, Hanne Scholz, Valdemar Grill

**Affiliations:** ^1^ Department of Clinical and Molecular Medicine Faculty of Medicine and Health Sciences Norwegian University of Science and Technology (NTNU) Trondheim Norway; ^2^ Department of Molecular Medicine and Surgery Karolinska Institutet Stockholm Sweden; ^3^ Department of Endocrinology St Olavs University Hospital Trondheim Norway; ^4^ Department of Transplantation Medicine Institute for Surgical Research Oslo University Hospital Oslo Norway

**Keywords:** Hyperoxia, insulin secretion, mitochondrial dysfunction, pancreatic beta cells, preterm birth

## Abstract

We previously showed that hyperoxia in vitro negatively affects beta cells of the rat. Here, we tested for possible clinical significance as well as mitochondrial interactions by hyperoxia, using human islets (function and viability), INS‐1 832/13 cells (mitochondrial metabolism), and mouse neonates (effects in vivo). Lastly, we assessed relevant parameters in a cohort of individuals born preterm and then exposed to hyperoxia. Human islets and INS‐1 832/13 cells were exposed to 24 h of hyperoxia (90–92% oxygen). Mouse neonates were subjected to 5 days of continuous hyperoxia. Individuals born preterm were evaluated in terms of glucose homeostasis and beta cell function by HbA1c and the HOMA2 formula. In human islets, hyperoxia significantly reduced glucose‐stimulated insulin secretion by 42.2 ± 5.3% and viability assessed by MTT by 22.5 ± 5.4%. Hyperoxia down‐regulated mitochondrial complex II by 21 ± 5% and upregulated complex III by 26 ± 10.1% and complex IV by 37 ± 10.6%. Partly similar effects on mitochondrial complexes were found in hyperoxia‐exposed INS‐1 832/13 cells. Exposure to hyperoxia swiftly reduced oxygen consumption in these cells and increased mitochondrial uncoupling. Hyperoxia transiently but significantly reduced insulin release in mouse neonates. Individuals born preterm displayed higher HbA1c versus controls, as well as insulin resistance. Thus, hyperoxia exerts negative effects in vitro on human beta cells and results indicate inhibitory effects on insulin secretion in vivo in mouse neonates. Negative effects may be lessened by the demonstrated swift and profound mitochondrial adaptability. Our findings open the possibility that hyperoxia could negatively affect beta cells of preterm human neonates.

## Introduction

Access to oxygen is essential for cells and tissues, including insulin‐producing beta cells (Dionne et al. 1993). A precarious balance exists between too little oxygen delivered (hypoxia) and too much (hyperoxia) (Kulkarni et al. 2007). Negative effects of hypoxia are well recognized and studied (Dionne et al. 1993; Cantley et al. 2010), however, those of hyperoxia are less so and their clinical importance have not been much elucidated.

We recently demonstrated that higher‐than‐normal oxygen environment during culture of rat pancreatic islets led to reduced insulin release, reduced oxygen consumption, other mitochondrial abnormalities and possibly also toxic effects (elevated ROS) following the hyperoxic period (Ma et al. 2014). These findings could have implications for in vitro pretransplantation protocols that aim to provide the best possible functional state for islets to be transplanted to diabetic individuals. Furthermore, since hyperoxia is part of the treatment of much preterm born babies, one should consider that such treatment could lead to impaired beta cell function during treatment and perhaps result in beta cell damage.

Against this background, this study was conceived to (1) assess the impact of hyperoxia on human beta cells in vitro. Upon finding major similarities of hyperoxia effects between human islets and clonal beta cells (INS‐1), we proceeded to use the cells to (2) gain insight into the time dynamics and mechanisms whereby hyperoxia affects mitochondrial function. Further, to assess an impact of hyperoxia in vivo (3) we tested for effects by hyperoxia in mouse neonates. Finally, we (4) assessed glucose homeostasis and beta cell function in a cohort of adults born preterm and treated with hyperoxia.

## Materials and Methods

### Materials

Mercaptoethanol was from VWR (Radnor, PA), Trypan blue was from Invitrogen (Carlsbad, CA) and DMSO from Merck Millipore (Darmstadt, Germany). Other materials were from Sigma‐Aldrich Chemical Co. (St Luis, MO) or from sources specified below.

### Methods

#### Procurement, isolation, and culture of human pancreatic islets

Human islets were isolated using a modified semi‐automated digestion method (Goto et al. 2004) from deceased donors at the islet isolation facility at the Section for Transplantation Surgery at Oslo University Hospital, Oslo, Norway, after appropriate consent was given for multi‐organ donation and for use in research. Islet preparations from six nondiabetic donors were used, two females and four males, aged 16–71 years with body mass index 20.9–30.5 kg/m^2^. The islet purity was 35–95% judged by digital imaging analysis or dithizone staining. Islet preparations used for research were restricted to those where limited quantity after isolations had precluded their use for clinical transplantation. Islet preparations were cultured in CMRL 1066 medium (Mediatech Inc., USA) supplemented with 10% human AB serum (Milan Analytica, Rheinfelden, Switzerland), 2 mmol/L l‐glutamine, 100 IU/mL penicillin and 100 *μ*g/mL streptomycin (all from Life Technologies). Upon arrival in Trondheim human islets were cultured free floating at 37°C at a humidified atmosphere of 5% CO_2_ in air in RPMI medium supplemented with 5.5 mmol/L glucose, 10% fetal calf serum, 1 mmol/L sodium pyruvate, 2 mmol/L l‐glutamine, 100 IU/mL penicillin and 100 *μ*g/mL streptomycin.

#### INS‐1 832/13 cells, basic conditions of culture

INS‐1‐derived 832/13 cells were developed by researchers now at Duke University (Hohmeier et al. 2000) and kindly provided by professor Hindrik Mulder, Malmö, Sweden. Cells were continuously tested and proven free from mycoplasma. Cells were grown in monolayer cultures in RPMI‐1640 medium with the same composition as for islets, except for the glucose concentration (11 mmol/L) and supplement with 50 *μ*mol/L mercaptoethanol. Cells were cultured at a humidified atmosphere of 5% CO_2_ in air. Cells were sub cultured once a week after detachment with 0.01% trypsin in 0.02% EDTA. The RPMI medium was changed every 3–4 days of culture.

#### Animals

Pregnant C576/6J mice were bought from Scanbur BK AB (Sollentuna, Sweden).

#### Experimental protocols

##### Human islets

**Figure 1:**
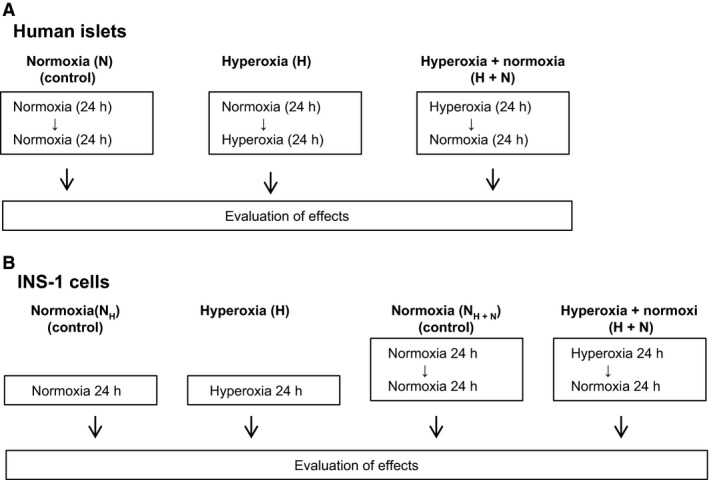
Experimental design. (A) Human islets and (B) INS‐1 832/13 cells were exposed to 24 h of hyperoxia. For the majority of experiments, effects of hyperoxia were evaluated directly after the hyperoxic event as well as after hyperoxia followed by 24 h of culture at normoxia. Control islets and cells were cultured in parallel at continuous normoxia.

The experimental protocol is depicted in Figure 1A. Islets were handpicked into each of three Petri dishes. In essence, two conditions of hyperoxia exposure were tested; in one the islets were exposed to 24 h of hyperoxia (representing the “hyperoxia” condition) and in the other islets were exposed to 24 h of hyperoxia followed by 24 h of normoxia (representing the “hyperoxia + normoxia” condition). Evaluation of hyperoxia‐induced effects on islets (for both conditions of hyperoxia exposure) was compared to those in control islets cultured in parallel for 48 h of normoxia.

Hyperoxia was induced by placing the islets in a closed chamber (Billups‐Rothenberg Inc., Del Mar, CA) together with an oxygen monitor and a Petri dish with 5 mL water to uphold humidity. The chamber interior was flushed with a mixture of 95% O_2_ and 5% CO_2_ until the desired O_2_ concentration was reached (90–92% O_2_).

##### Ins‐1 832/13

For ATP quantification and oxygen consumption experiments cells were seeded in 25 cm^2^ culture flasks. For other experiments, cells were seeded in multi‐well plates. Cells (passage 46–66) were cultured for 3 days before exposure to hyperoxia for 24 h, in some protocols with, in other without a subsequent 24 h period of normoxia (Fig. 1). Hyperoxia was induced as for human islets. To ensure equal confluence at the time of exposure to hyperoxia, hyperoxia was always performed on day 4 after seeding. Evaluation of hyperoxia‐induced effects in cells exposed to 24 h of hyperoxia (the “hyperoxia” condition) were compared to those in control cells cultured in parallel for 24 h of normoxia. Correspondingly, effects of 24 h of hyperoxia followed by 24 h of normoxia (the “hyperoxia + normoxia” condition) were compared to control cells cultured in parallel for 48 h at normoxia. The experimental protocol is shown in Figure 1B.

Acute ambient hyperoxia was induced by flushing the surface of the cell suspension in the Oxygraph chamber (see below (HRR)) with a 95% O_2_ and 5% CO_2_ mixture until an O_2_ concentration of 3–400 *μ*mol/L was reached. The chamber was then closed and oxygen consumption measurements started.

##### Mouse animal model

Pups were divided into two groups 14 days after delivery. Each group consisted of 7–10 pups who resided in one or two litters. One group (control) was kept under normal oxygen conditions. The other group was subjected to 70% oxygen for 5 days inside a closed chamber where the gas volume was replenished three times per 24 h. The temperature was regulated to between 20 and 22°C and was similar for the hyperoxia‐exposed and the control group. No deaths occurred before or during the 5 day intervention period. During this period, lactating mothers were switched daily between the hyperoxia‐exposed and the control litter (in order to avoid any bias from a possible influence of hyperoxia on lactation). At the end of the 5 day period with or without hyperoxia, the pups were checked for body weight and blood glucose. After sacrifice pancreatic islets were isolated by injection of collagenase into the pancreatic duct under microscope (Ohki et al. 2012) and cultured overnight in RPMI medium containing 11 mmol/L glucose.

Following the same protocol with regard to age and duration of exposure to hyperoxia some mice were kept alive to 10 weeks of age at which time an i.p. glucose tolerance test was performed. The test was performed in the non‐fasted state and without anesthesia. Blood samples were obtained from a tail vein.

#### Cell counting

INS‐1 832/13 cells were seeded either in 25 cm^2^ culture flasks or on to 24 well plates and cultured as detailed above. For cell counting Trypan blue stain (0.4%) and a sample of the cell suspension were mixed (1:1) and viable and dead cells counted in a Countess automatic cell counter (Invitrogen, Carlsbad, CA).

#### Apoptosis/necrosis

##### Human islets

Samples for necrotic and apoptotic DNA fragment quantification were secured directly after the period of hyperoxia as well as after another 24 h culture at normoxia (the “hyperoxia + normoxia” condition). Necrotic and apoptotic DNA fragments were quantified by the Cell Death Detection ELISA^plus^ kit (Roche Diagnostics Gmbh, Mannheim, Germany).

#### MTT

##### Human islets

For the 3‐(4,5‐diethylthiazol‐2‐yl)‐2,5‐diphenyltetrazolium bromide (MTT) assay (Mosmann 1983) islets were handpicked into Petri dishes for exposure to either 24 h of hyperoxia, 24 h of hyperoxia followed by 24 h of normoxia, or to continuous normoxia. Islets were then transferred to a 24 well plate (30–40 islets/well, 2–4 parallels for each experimental condition) for 4 h of MTT exposure. The MTT reagent in the media was removed by washing the islets several times in 0.9% NaCl. Islets were then incubated for 1 hour in 400 *μ*L DMSO per well at 37°C for color development. Fifty *μ*L/well of 0.1 mol/L NaCl in 0.1 mol/L Glycin, pH 10.5, was added for color extraction. Two parallel aliquots per well were secured for absorbance measurements.

##### INS‐1 832/13 cells

INS‐1 832/13 cells were seeded in 96‐well plates (10^4^ cells/well) and cultured as described above (Experimental conditions). The MTT assay was performed either directly after the hyperoxia exposure or, in separate experiments, after normoxia (the “hyperoxia + normoxia” condition). After 4 h of MTT exposure the MTT reagent was removed, followed by a 1 h exposure to isopropyl alcohol.

#### Insulin secretion and content

##### Human islets

Islets were first exposed to 24 h of hyperoxia – which in a subset of experiments hyperoxia was followed by 24 h of normoxia. Islets were then preincubated for 30 min (in Petri‐dishes) in KRB (containing 0.5% BSA and 10 mmol/L Hepes at pH 7.4) together with 1.6 mmol/L glucose. For each experimental condition, groups of 4 islets were placed in each of 5–8 parallel tubes and incubated for another 60 min in KRB (0.3 mL/tube) containing 1.6 mmol/L glucose (basal secretion). KRB was then removed, new KRB containing 16.7 mmol/L glucose (0.3 mL/tube) was added and the islets incubated for a further 90 min. Incubation media were secured for both basal and stimulated secretion. At the end of experiments, the content of insulin from the islets was extracted overnight at 4°C after sonication for 10 sec in acid‐ethanol (580 *μ*L HCl in 50 mL 95% ethanol), 0.2 mL/tube. Samples were kept at −20°C pending insulin measurements by RIA kit for human insulin (Millipore, St Charles, MO).

##### INS‐1 832/13 cells

Cells were seeded in 24‐well plates (10^5^ cells per well). After 3 days of culture, the medium was renewed in preparation for cells being exposed to hyperoxia for 24 h without or – in separate experiments – with an ensuing period of normoxia (the “hyperoxia + normoxia” condition). Control cells were cultured under normoxic conditions. Prior to glucose stimulation the RPMI medium was removed and cells were cultured for a further 2–3 h in RPMI modified to contain no glucose and supplemented with 20 mmol/L Hepes and 1% FCS. Cells were then preincubated for 30 min in Krebs‐Ringer bicarbonate‐HEPES buffer (KRBH) with 10 mmol/L HEPES, 0.1% BSA and in the absence of glucose. Final incubations were carried out in 0.5 mL of KRBH per well for 60–90 min in media containing 3.3, 11 or 27 mmol/L glucose (3 parallels per condition). Aliquots of media were secured for insulin assay. A glucose stimulation index (GSI) was calculated by dividing the insulin (*μ*U/well) that had accumulated during exposure to 11 and 27 mmol/L by the insulin accumulated during exposure to 3.3 mmol/L glucose. Intracellular insulin was extracted overnight at 4°C after adding acid‐ethanol, 0.5 mL/well. Immunoreactive insulin was analyzed by RIA (Herbert et al. 1965).

##### Mouse animal model

Islet isolated during the day of sacrifice were cultured overnight in RPMI medium with the composition indicated above. Aliquots from these culture media were secured for insulin assay. Equal‐sized islets were then preincubated the day after as previously reported (Bjorklund and Grill 1993), followed by a 60‐min batch incubation (3 islets per tube in triplicate, 2.8 or 16.7 mmol/L glucose). Aliquots of incubation media were secured for measurements of secreted insulin. Islets from the batch incubations were also secured to extract cellular insulin (as described for human islets). Immunoreactive insulin was analyzed by RIA (Herbert et al. 1965).

#### ATP quantification

The ATP Bioluminescence Assay Kit CLS II (Roche Diagnostics GmbH, Mannheim, Germany) was used for the quantification of ATP in INS‐1 832/13 cells. Samples were diluted to a concentration of 10^6^ cells/mL and the assay was performed as recommended by the producer.

#### Western blotting

##### Human islets

Islets were washed twice in ice‐cold PBS and lysed on ice for 20–30 min in lysis buffer (1 *μ*L lysis buffer per 5 islets). The lysis buffer contained 150 mmol/L NaCl, 50 mmol/L Tris‐HCl (pH 7.4), protease inhibitor cocktail (Complete Mini, Roche Diagnostics Gmbh, Mannheim, Germany), 1% Nonidet P40 (NP‐40), 10% glycerol, 50 mmol/L NaF and 1 mmol/L Na_3_VO_4_. The Micro BSA™ Protein Assay Kit (Thermo Scientific, Rockford, IL, USA) was used for the quantification of protein in the lysates.

Protein samples were denatured in loading buffer for 20 min at room temperature and further analyzed on 12% SDS‐PAGE gels run for 1 h at 150 V. Samples were then transferred to nitrocellulose membranes for 1 h at 250 mA. Membranes were blocked for 2 h at room temperature with 5% (w/v) fat‐free milk, 0.1% Tween 20 in Tris‐buffered saline, pH 7.6 and then incubated over night at 4°C with Total OXPHOS Rodent WB antibody Cocktail (1:5000, MitoSciences, Eugene, OR, USA). Monoclonal mouse anti‐beta‐actin (Sigma) was used as loading control. Secondary antibody incubations employed a HRP‐linked anti‐mouse antibody for 1 h at room temperature. Immunoreactive bands were visualized using chemiluminescence (ECL Western blotting reagent, Pierce, Biotechnology, USA). Primary antibodies were used at 1:500 dilutions for oxidative phosphorylation complexes 1–4 MS604 (MioSciences, USA).

##### Cells

Cells were trypsinized and washed two times in ice‐cold phosphate‐buffered saline (PBS). Cells (5 × 10^5^) were lysed on ice for 20–30 min in 20 *μ*L lysis buffer and further analyzed as described for human islets.

#### High resolution respirometry General

Respirometric measurements were done by the Oxygraph‐2k (OROBOROS, Innsbruck, Austria) instrument which makes use of Clark polarographic oxygen sensors. All calibrations and experiments were performed at 37°C with magnetic stirring set at 750 rpm, Chemicals for oximetry that were used in the respirometric protocols were as described (Hals et al. 2015). They were prepared, pH‐adjusted and stored as recommended by OROBOROS INSTRUMENTS. Oxygen concentrations (nmol/mL) in the chambers containing the samples were measured and analyzed by the Datlab software (OROBOROS INSTRUMENTS). Oxygen flux (pmol O_2_/s/10^6^ cells) was calculated as the negative time derivative of measured oxygen concentration.

##### In protocols measuring oxygen consumption in intact cells

Two mL samples of an INS‐1 832/13 cell suspension (10^6^ cells/mL in RPMI culture media) were added to the Oxygraph chambers. For protocols in which cells were permeabilized, the cell suspension was first re‐centrifuged and the cell pellet re‐suspended in the mitochondrial respiration medium MiR05, composition given below, (10^6^ cells/mL) before being added to the Oxygraph chambers.

For experiments testing effects of acute ambient hyperoxia, in six of the experiments, one chamber was flushed with a 95% O_2_ and 5% CO_2_ mixture and the other with air (21% O_2_). In the other 14 experiments the chamber with hyperoxia was included in an unrelated protocol (therefore significance testing was done for unpaired observations). For experiments with cells tested after exposure to 24 h of hyperoxia we dispensed aliquots from each sample into each of the two chambers. The results from the duplicates were then averaged before calculations.

##### In protocols using permeabilized Cells

INS‐1832/13 cells the mitochondrial respiration medium MiR05 was used. MiR05 included EGTA (0.5 mmol/L), MgCl_2_·6 H_2_O (3 mmol/L), lactobionic acid (60 mmol/L), taurine (20 mmol/L), KH_2_PO_4_ (10 mmol/L), HEPES (20 mmol/L), D‐Sucrose (110 mmol/L) and BSA, essentially fatty acid free (1 g/L). The pH was adjusted to pH 7.1 as recommended by OROBOROS INSTRUMENTS.

Two mL samples of cell suspension was added to the Oxygraph chamber (10^6^ cells/mL) and exposed to acute ambient hyperoxia (see Experimental protocols). Signals with cells (still intact) were allowed to stabilize before the subsequent addition of digitonin (8.1 *μ*mol/L, 10 *μ*g/10^6^ cells). The optimal digitonin concentration for permeabilization of the cells was found in exploratory experiments (Hals et al. 2015). Stable respiration in the presences of digitonin was awaited before continuing with the experimental protocol. Based on previous experience (Hals et al. 2015) glutamate (10 mmol/L) was chosen as the sole substrate for complex I.

#### Cohort study design

We used data from three groups of neonates born during 1986–88 at St. Olavs Hospital in Trondheim. In one group, neonates were born preterm (<37 gestational week, median gestational week: 29) with very low birth weight (VLBW, birth weight ≤1500 g) and therefore treated with hyperoxia during intensive care. Another group comprised individuals born at term (37–42 gestational week, median gestational week: 39) but small for gestational age (SGA, birth weight <10th centile adjusted for gestational age, sex and parity). A third group constituted individuals born at term (median gestational week: 40) with normal weight (birth weight > 10th centile). Several publications have reported results from these cohorts (Evensen et al. 2009; Lohaugen et al. 2010).

Serum samples and data on fasting glucose and HbA1c from the participants (collected between June 2013 and September 2014) were available from a recent follow‐up of the cohorts (Balasuriya et al. 2017). Fasting serum C‐peptide levels were measured by RIA kit for human C‐peptide (Millipore, St Charles, MO).

#### HOMA calculations and assessment of beta cell function

Calculations of the Homeostatic Model Assessment 2 (HOMA2) for insulin resistance (IR), beta cell function (%B) and insulin sensitivity (%S) (The Oxford Center for Diabetes, 2013) were based on levels of fasting C‐peptide and fasting glucose. Data from 55 individuals with VLBW, 62 SGA and 74 controls were included in the calculations.

### Ethics

Human pancreata were obtained from non‐diabetic brain‐dead donors provided by the isolation facility of the Oslo University Hospital, Oslo, Norway, after verbal informed consent from relatives for multi‐organ donation and for use in research. All experiments and methods using human islets were approved by and performed in accordance with the guidelines and regulations made by regional committee for medical and health research ethics central in Norway (2011/782).

For the mouse animal study the Ethical Guidelines of the Karolinska Institutet for the care and use of laboratory animals were followed (permit number N145/14).

The cohort study was approved by the Regional Committee for Medical and Health Research Ethics in Central Norway (permit number 2013/636/REK Mid‐Norway). All participants gave written informed consent.

### Statistics

All data are presented as mean ± SEM. For experiments with human islets, INS‐1 cells and the animal study, significant differences were tested using the Student's *t*‐test (paired or unpaired), the Wilcoxon rank test or the Mann–Whitney test as appropriate. For the comparisons of HOMA2 parameters from the cohort study the Kruskal–Wallis test was used. A *P*‐value <0.05 was regarded as significant.

## Results

### Hyperoxia in vitro impairs function and viability of human islets

**Figure 2:**
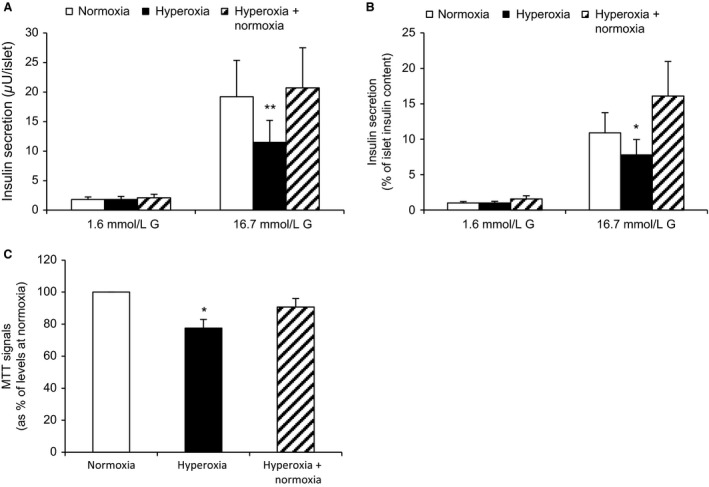
Hyperoxia reduces insulin secretion and viability in human islets. Insulin secretion is in (A) expressed as *μ*U/islet and in B) as % of islet insulin contents. Glucose‐induced insulin secretion (GSIS) was reduced after exposure to 24 h of hyperoxia, **P < 0.005 versus controls for *μ*U/islet and *P < 0.05 for % of islet insulin contents, *n* = 10 (2–5 experiments per donor, three donors). For half of the 10 experiments, measurements were performed also after hyperoxia followed by normoxia (2–3 experiments per donor, 2 donors). (C) Viability assessed by MTT was reduced by hyperoxia, *P < 0.02 for difference versus control, *n* = 10 (1–4 experiments per donor, four donors). Statistics were performed using Wilcoxon paired rank test.

Glucose‐induced insulin secretion (*μ*U/islet) was reduced by 42.2 ± 5.3% (*P* < 0.005, based on 10 separate experiments from three donors. For calculations based on the number of donors, *n* = 3, the reduction was 40.4 ± 7.1%, *P* < 0.11) following 24 h of culture during hyperoxia (Fig. 2A). When hyperoxia was succeeded by a 24 h period of normoxia, the negative effects on secretion were absent. The total amount of islet insulin contents were not affected by hyperoxia (227.2 ± 44.1 *μ*U/islet after hyperoxia vs. 239.4 ± 37.6 *μ*U/islet at normoxia, i.e. 95.5 ± 9.9% of levels at normoxia, in ten separate experiments from three donors). Effects of hyperoxia on the viability of human islets were tested by MTT (Fig. 2C). The MTT signal vas reduced by 22.5 ± 5.4% (*P* < 0.02, based on ten separate experiments from three donors. For calculations based on the number of donors, *n* = 3, the reduction was 17.3 ± 6.3%, *P* < 0.07) following hyperoxia. No significant effect on MTT remained when hyperoxia was succeeded by 24 h of normoxia. Islet death was evaluated by measurements of apoptotic and necrotic DNA. No effects of hyperoxia on these parameters were detected (results not shown).

### Previous hyperoxia affects mitochondrial complexes of human islets

**Figure 3:**
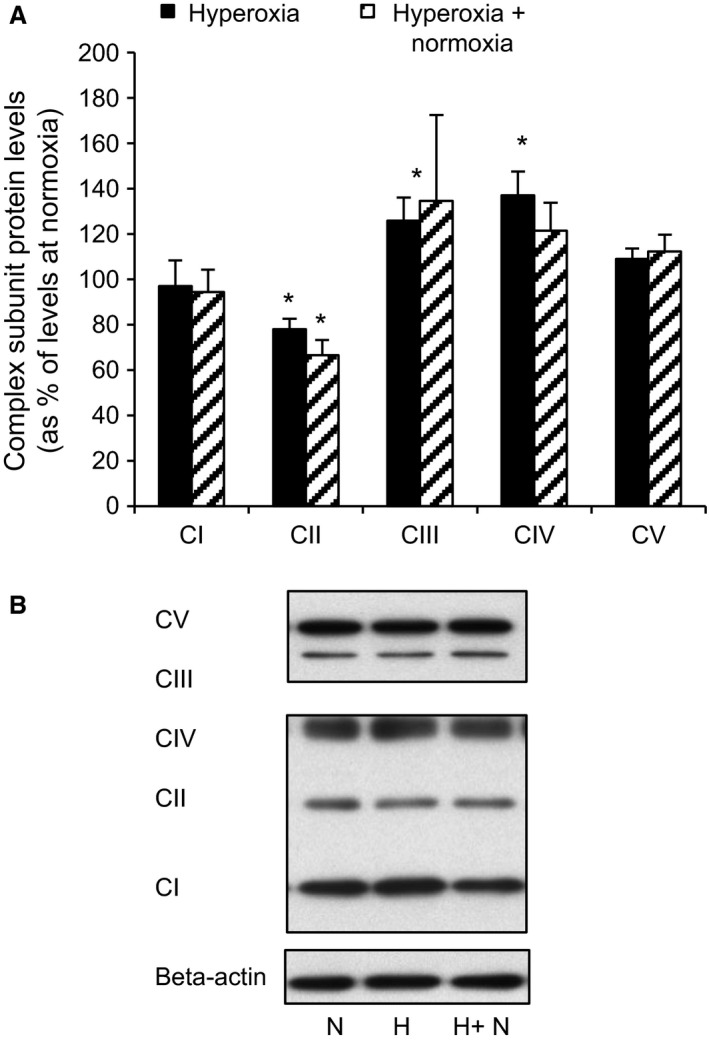
Hyperoxia affects mitochondrial complex protein levels in human islets. (A) shows protein levels of complex (C) *I*–*V* after previous hyperoxia, expressed as percentage of corresponding protein levels in control islets (normoxia). *P < 0.02 for effects of previous hyperoxia (either with or without a 24 h period of normoxia) versus normoxia, from *n* = 7 (CV) or 9 (CI‐IV) separate experiments from 4 (CV) or 5 donors (CI‐IV). Statistics were performed using Wilcoxon paired rank test. (B) shows a representative Western blot for levels of complexes *I–V* in human islets at normoxia, after 24 h of hyperoxia and after 24 h of hyperoxia followed by 24 h of normoxia. The abbreviations used correspond to the experimental protocols as shown in Fig. 1. Band images were from separate parts of the same gel.

To test for mitochondrial adaptions to hyperoxia we measured protein components of mitochondrial complexes *I*–*V* in human islets (Fig. 3A, summary of results and B, a typical Western blot). Following 24 h of hyperoxia complex II was significantly reduced (by 22 ± 4.7%, *P* < 0.02, *n* = 9 separate experiments from five donors, Figure 3A. For calculations based on the number of donors, *n* = 5, the reduction was 24 ± 2.7%, *P* < 0.05). The reduction persisted after the subsequent exposure to 24 h of normoxia (33.5 ± 6.8% reduction vs. levels at normoxia, *P* < 0.02, *n* = 9. For calculations based on the number of donors the reduction was 32.8 ± 7.6%, *P* < 0.05). Following 24 h of hyperoxia complex III and IV were significantly increased (by 26 ± 10.1% and 37 ± 10.6% vs. levels at normoxia, *P* < 0.02, *n* = 9. Corresponding values calculated on the basis of the number of donors were 35.6 ± 16.2% and 39.4 ± 14.8%, *P* < 0.05). No significant reductions remained when hyperoxia was followed by 24 h of normoxia.

### Effects of hyperoxia on human islets can be partially reproduced in clonal beta cells

**Figure 4:**
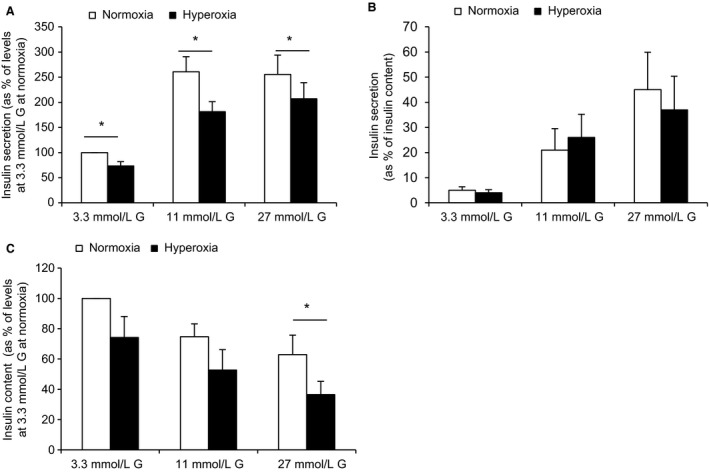
Hyperoxia reduces insulin secretion and insulin contents in INS‐1 cells. (A) and (B) show insulin release at 3.3, 11 and 27 mmol/L glucose (G), in (A) expressed as percent of levels at 3.3 mmol/L glucose (G) at normoxia and in (B) relative to cellular insulin contents. (C) shows insulin contents. *P < 0.05, *n* = 6. Statistics were performed using Wilcoxon paired rank test.

**Figure 5:**
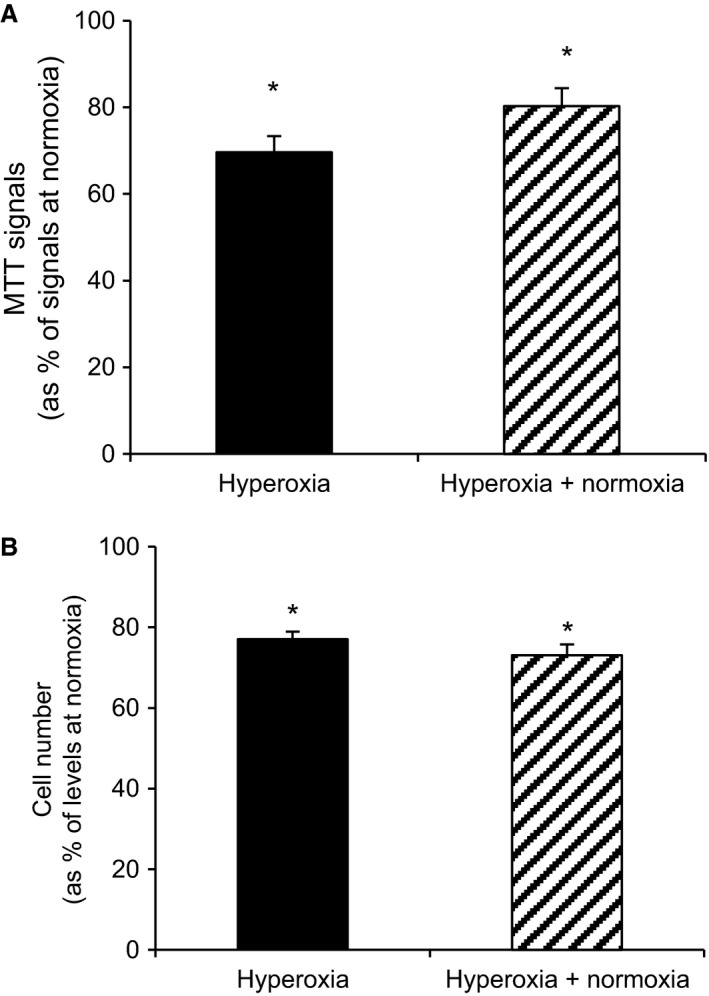
Effects of hyperoxia on INS‐1 cell viability. Viability was assessed by (A) MTT (*n* = 6–7) and (B) cell counting (*n* = 6–9). *P < 0.03 for differences versus normoxia. Statistics were performed using Wilcoxon paired rank test.

**Figure 6:**
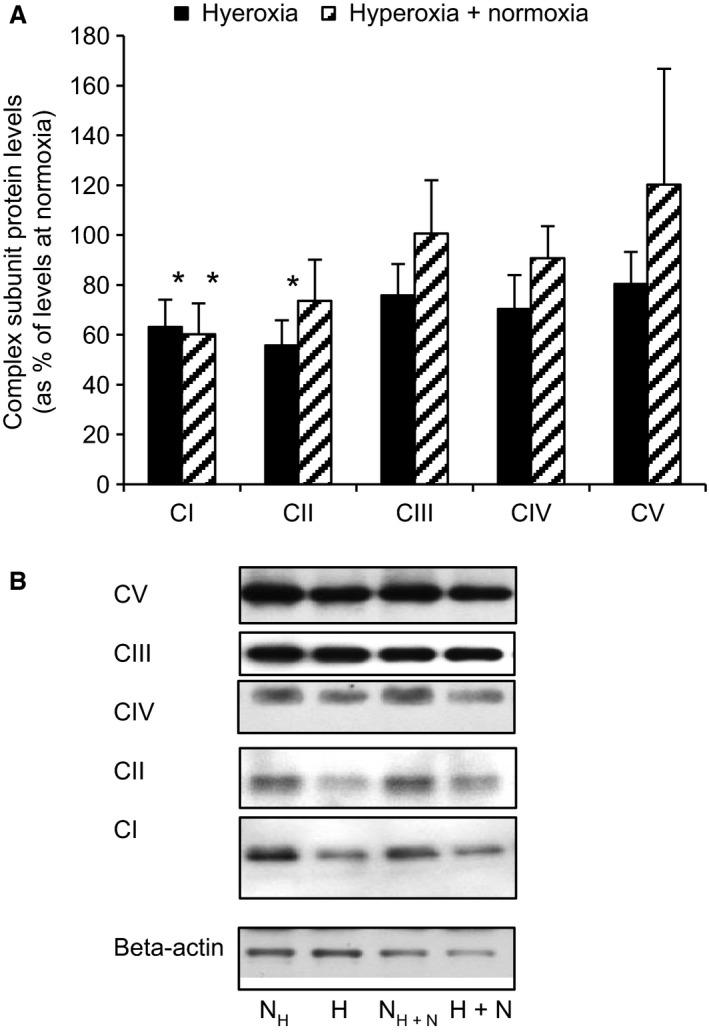
Hyperoxia affects mitochondrial complex protein levels in INS‐1 cells. (A) shows protein levels of complexes (C) *I–V* after previous hyperoxia, expressed as percentage of corresponding protein levels in control islets (normoxia), *P < 0.05 for differences versus normoxia, *n* = 6. Statistics were performed using Wilcoxon paired rank test. (B) Representative Western blot for levels of complexes *I–V* in INS‐1 cells after 24 h of normoxia) versus levels after 24 h of hyperoxia and after 48 h of normoxia versus 24 h of hyperoxia followed by 24 h of normoxia. The abbreviations used correspond to the experimental protocols as shown in Figure 1. Band images were from separate parts of the same gel.

As a prerequisite for further mechanistic studies on hyperoxia using clonal beta cells we tested for hyperoxia effects in INS‐1 cells. Exposure to hyperoxia for 24 h decreased both insulin secretion (Fig. 4A) and cellular insulin contents (Fig. 4C). Thus when expressing results in per cent of insulin contents no difference was found between hyperoxia‐cells versus control cells (Fig. 4B). The same effects of hyperoxia were apparent also when hyperoxia was followed by 24 h of culture at normoxia (in six separate experiments, results not shown). Cell viability was tested by MTT and cell counting (Fig. 5). For both these parameters there was a significant reduction after 24 h of hyperoxia, an effect that was present also after a subsequent 24 h of culture at normoxia. The MTT signals were reduced by 30.4 ± 3.7% (*P* < 0.03, *n* = 7) after hyperoxia and by 19.7 ± 4.1% (*P* < 0.03, *n* = 6) after hyperoxia + normoxia. Correspondingly the number of cells per culture flask were reduced by 22.9 ± 2.7% (*P* < 0.01, *n* = 9) and 26.9 ± 5.5% (*P* < 0.03, *n* = 6). Proteins of complexes I and II of remaining cells were decreased after 24 h of hyperoxia by 36.9 ± 11.0 and 44.3 ± 10.1% (*P* < 0.05, *n* = 6), respectively. For complex I the reduction lingered as tested after an additional 24 h at normoxia (39.8 ± 12.4%, *P* < 0.05, *n* = 6) (Fig. 6A, summary of results and B, a typical Western blot).

### Hyperoxia reduces mitochondrial oxygen consumption and swiftly increases uncoupling

**Figure 7:**
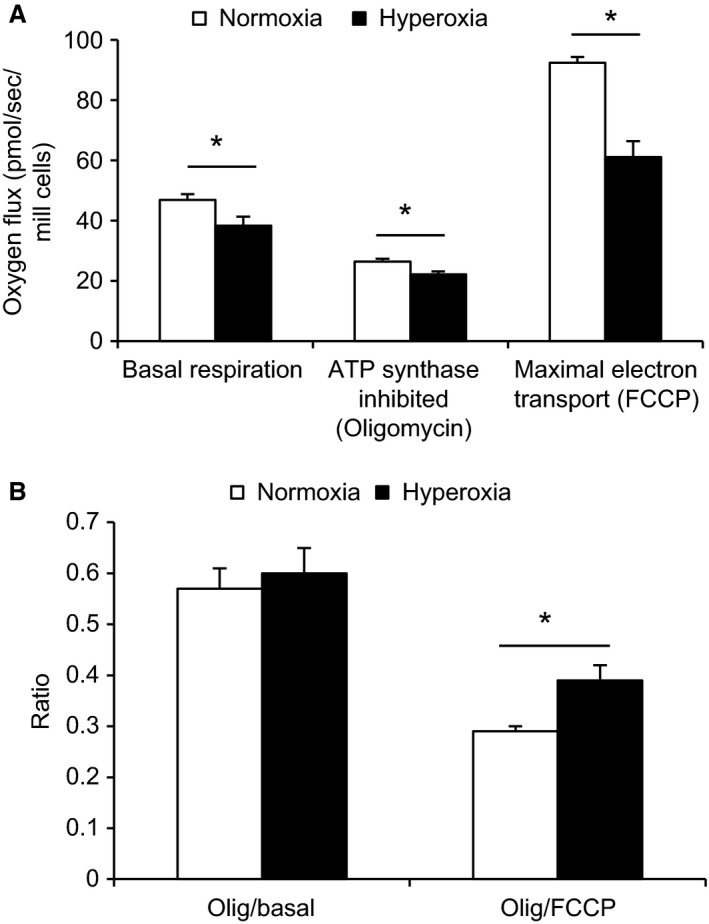
Effects of 24 h of hyperoxia on oxygen consumption in INS‐1 cells. (A) Respiration at basal conditions, after inhibition of ATP synthase by oligomycin (uncoupled respiration) and at the fully uncoupled state achieved by FCCP. *P < 0.0002–0.05 by Student's unpaired *t*‐test, *n* = 7–8. (B) An increased ratio of residual oxygen consumption (after oligomycin) to maximal oxygen consumption (after FCCP) indicates increased uncoupling. *P < 0.01 by the Mann–Whitney test, *n* = 7–8.

Following 24 h of hyperoxia, basal respiration as well as ATP synthase inhibited (by oligomycin) and fully uncoupled respiration (by FCCP) was significantly decreased in intact INS‐1 cells (Fig. 7A). Basal oxygen consumption was decreased from 46.9 ± 1.9 to 38.4 ± 2.9 pmol/s/10^6^ cells and, after the introduction of oligomycin, from 22.2 ± 1.0 to 26.4 ± 1.0 pmol/s/10^6^ and after FCCP from 92.5 ± 1.9 to 61.1 ± 5.3 pmol/s/10^6^ (*P* < 0.0002–0.05, *n* = 7–8). Furthermore, the ratio of residual oxygen consumption (after oligomycin) to maximal oxygen consumption (after FCCP) was increased (*P* < 0.01, *n* = 7–8), indicating increased uncoupling (Fig. 7B).

**Figure 8:**
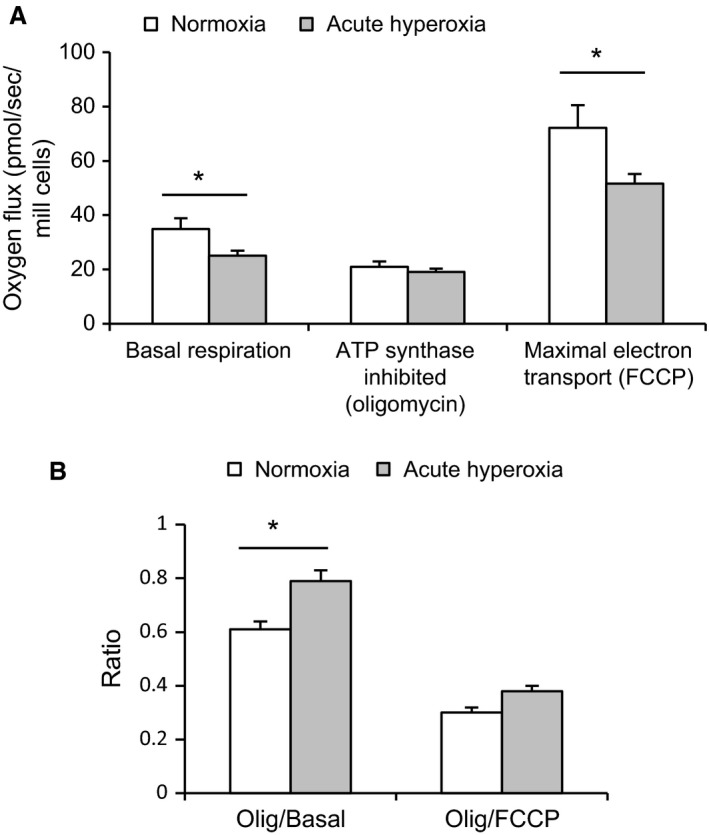
Effects of acute ambient hyperoxia on oxygen consumption in INS‐1 cells. Respiration in INS‐1 cells during exposure to acute hyperoxia versus normal air (normoxia). (A) Oxygen consumption is depicted at basal conditions, then after inhibition of ATP synthase by oligomycin (uncoupled respiration) followed by the fully uncoupled state achieved by FCCP. (B) Increased ratios of residual oxygen consumption (after oligomycin) to basal respiration and to maximal oxygen consumption (after FCCP,* P* < 0.06) indicate increased uncoupling at acute hyperoxia. *P < 0.03, *n* = 6 (normoxia) and 20 (hyperoxia). Statistics were performed using the Student's unpaired *t*‐test (A) and the Mann–Whitney test (B).

To investigate ambient effects of hyperoxia on oxygen consumption we measured consumption during the presence of hyperoxia (in the reaction chamber) in the oxygraph. Compared to the response to oligomycin in cells at normoxia, cells exposed to acute hyperoxia appeared to a lesser extent affected by the inhibition of ATP synthase (Fig. 8A). This was indicated by a significant increase in the ratio of residual respiration (after oligomycin) to basal respiration (Fig. 8B), which would amount to ≈20% of basal oxygen consumption being coupled to ATP synthesis during acute hyperoxia versus ≈40% at normoxia (*P* < 0.03, *n* = 6–20). Increased uncoupling was also indicated by an increase in the ratio of residual oxygen consumption to maximal oxygen consumption (*P* < 0.06, *n* = 6–20) (Fig. 8B). Remarkably, in this protocol hyperoxia had been present for only 20–30 min before the respiratory response to oligomycin was altered.

### Effect of acute hyperoxia on respiration in permeabilized cells

**Figure 9:**
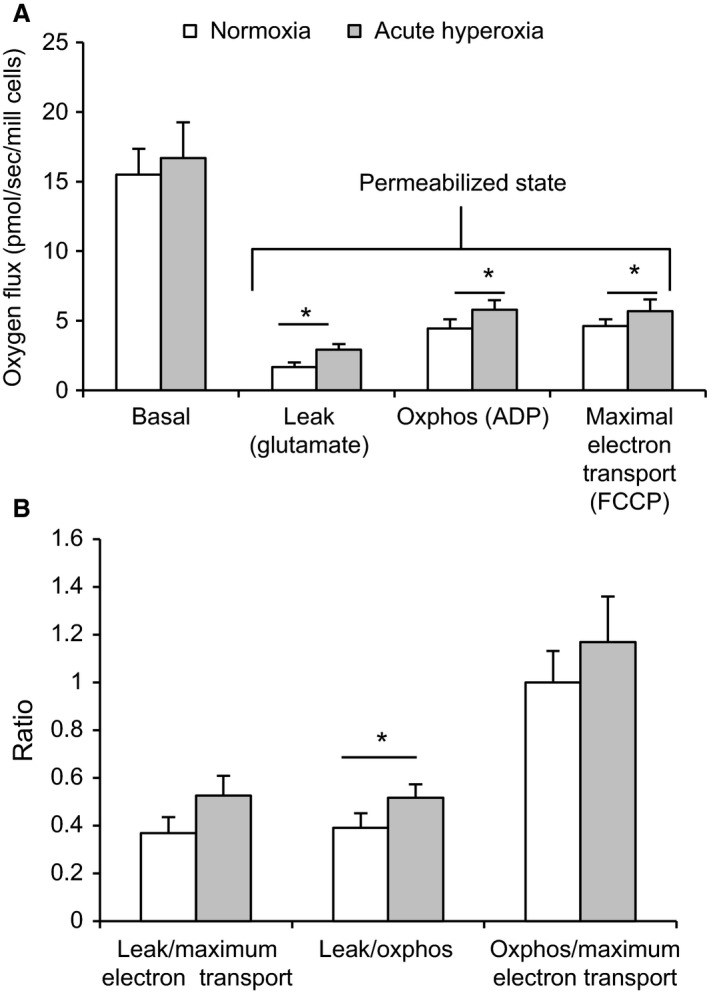
Effects of hyperoxia on oxygen consumption in permeabilized INS‐1 cells. (A) shows oxygen consumption in intact cells at basal conditions in MiR05 media (basal respiration), in permeabilized cells after adding glutamate (Leak), after adding ADP (Oxphos) and at the fully uncoupled state by FCCP (ETS). (B) An increased ratio of Leak to Oxphos at acute hyperoxia versus normoxia indicates increased uncoupling. *P < 0.04, *n* = 9. Statistics were performed using the Student's unpaired *t*‐test and (A) and Wilcoxon rank test (B).

Respiration in permeabilized cells was tested in relation to the complex I substrate glutamate. In the absence of ADP (state 2) hyperoxia increased respiration, an observation which would be compatible with an uncoupling effect (Fig. 9). Oxygen consumption was moderately increased in state 3, that is, after the addition of ADP.

### Previous hyperoxia did not affect levels of cellular ATP

ATP contents in INS‐1 cells after 24 h of hyperoxia exposure were 90.2 ± 7.0% of contents in control cells (*n* = 6). The corresponding levels in cells exposed to hyperoxia followed by 24 h of normoxia were 98.3 ± 13.6% (*n* = 6).

### Hyperoxia in vivo: negative effects in a mouse model

**Figure 10:**
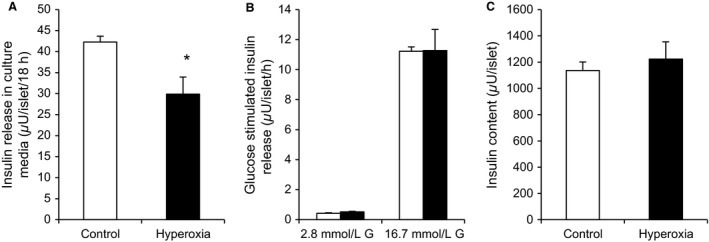
Effect of hyperoxia in vivo on mouse neonates. Islets were isolated from hyperoxia‐exposed pups (5 days at 75% oxygen) (black bars) and controls (white bars). (A) Insulin release to the culture media during the first 24 h after the exposure, *P < 0.05, *n* = 3. Statistics were performed using the Mann–Whitney test. Subsequent measurements showed no effects of the hyperoxia treatment on (B) insulin secretion in batch incubations at 2.8 mmol/L and 16.7 mmol/L glucose (G) nor on (C) islet insulin contents.

The in vitro studies did not answer the question whether and to which extent hyperoxia in vivo could affect beta cells. To address this question we used an animal model. Islets were isolated from mouse pups after 5 days of exposure to 75% oxygen. Insulin release from these islets was decreased during the first 24 h of culture following the hyperoxic exposure (Fig. 10A). The decrease amounted to 27.3 ± 10.8% (*P* < 0.05 for *n* = 3 separate experiments, 28.4 ± 5.9%, *P* < 0.002, for the total number of pups exposed (*n* = 16) to hyperoxia or not (*n* = 16)). The effect was transient, since a later test for glucose stimulation of insulin release did not reveal any abnormality (Fig. 10B), nor were insulin contents affected (Fig. 10C). Exposure to hyperoxia did not significantly affect body weight (7.4 ± 0.4 g for hyperoxia treated neonates vs. 7.6 ± 0.6 g for controls, *n* = 3 separate experiments) nor blood glucose levels (6.8 ± 1.0 mmol/L for hyperoxia treated neonates vs. 8.4 ± 0.2 mmol/L for controls, *n* = 3 separate experiments). Also in an i.p. glucose tolerance test in 10‐week‐old mice, performed in 7 control and 9 previously hyperoxia‐exposed mice, results did not reveal any effect of previous hyperoxia. Hence, blood glucose before the test was in controls 4.7 ± 0.09 mmol/L and in hyperoxia‐exposed mice: 5.5 ± 0.39 mmol/L. Blood glucose at the end of the 120 min test was in controls 6.3 ± 0.31 mmol/L and in hyperoxia‐exposed mice: 6.5 ± 0.29 mmol/L. Also, weights were similar (20.4 ± 3.09 g for controls, 21.0 ± 2.13 g for hyperoxia‐treated).

### HbA1c and HOMA parameters

Levels of HbA1c were slightly but significantly higher in the VLBW group versus controls (5.21 ± 0.03 vs. 5.09 ± 0.03, *P* < 0.005). Levels tended to be higher also in the SGA group versus controls (5.17 ± 0.03 vs. 5.09 ± 0.03, *P* < 0.058). C‐peptide levels were modestly increased in the VLBW group versus controls (0.73 ± 0.04 vs. 0.63 ± 0.03, *P* < 0.04, using log transformation and ANOVA with Bonferroni).

**Figure 11:**
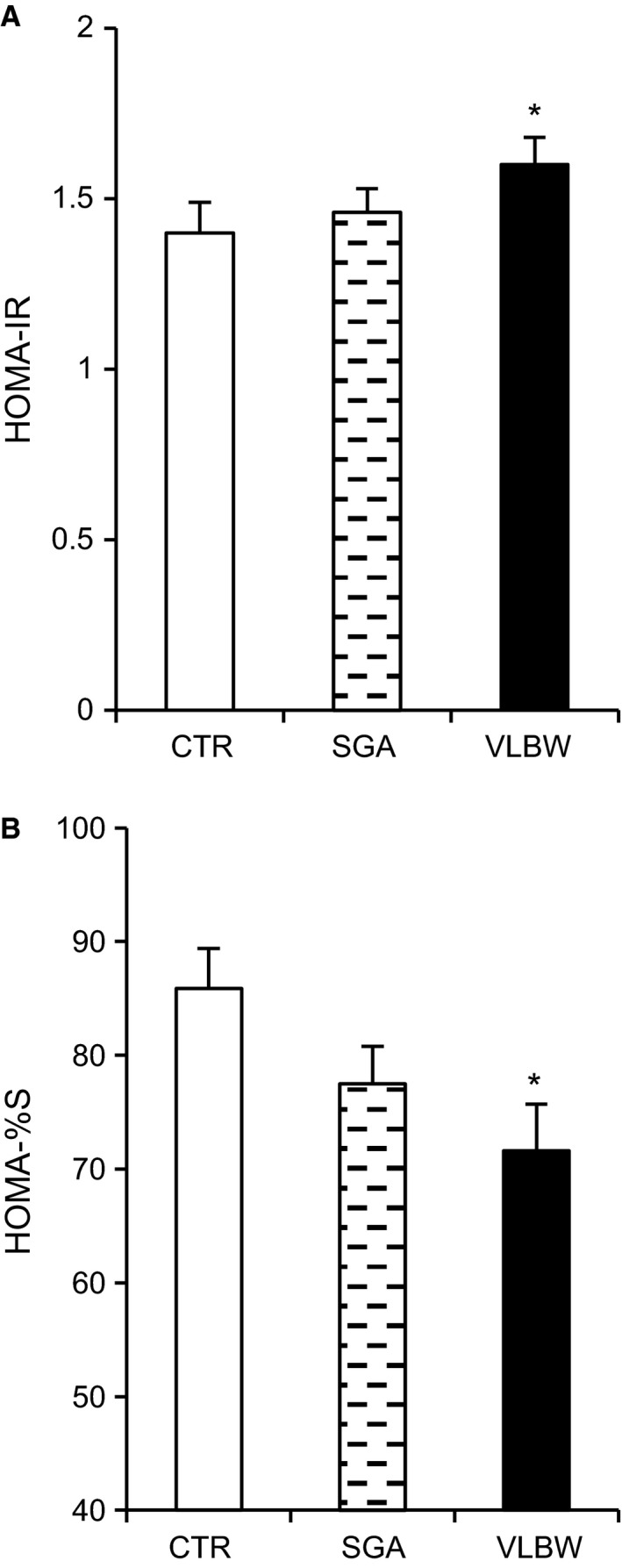
Evaluation of insulin resistance and beta cell function by HOMA in adults born preterm. (A) Insulin resistance (HOMA2‐IR), (B) beta cell function (HOMA2‐%B) and insulin sensitivity (HOMA2‐%S). *P < 0.016 for difference versus controls, using the Kruskal–Wallis test.

For individuals in the VLBW group versus the control group, HOMA parameters indicated increased insulin resistance by increased HOMA‐IR (1.61 ± 0.09 vs. 1.40 ± 0.08, *P* < 0.016) and reduced HOMA‐%S (71.6 ± 3.5 vs. 85.9 ± 4.1, *P* < 0.016), a measure of insulin sensitivity. There was also a tendency for increased beta cell capacity in the VLBW group versus controls (HOMA‐%B: 131 ± 4.9 vs. 122 ± 4.1, *P* < 0.072) (Fig. 11).

## Discussion

A principal aim of this study was to test whether effects of hyperoxia in vitro in human islets were akin to the negative effects, which were previously demonstrated in rat islets (Ma et al. 2014). The affirmative findings we obtained in human islets in vitro motivated us to (1) elucidate interactions between hyperoxia and mitochondrial function and (2) verify an influence of hyperoxia also in vivo using an animal model. Lastly, we had the opportunity to assess glucose homeostasis and insulin secretion in a cohort of adults born preterm and exposed to hyperoxia during the preterm period of life.

The results in human islets thus demonstrate negative effects by hyperoxia, effects that appear similar to those previously reported in rat islets. As in rat islets, previous hyperoxia impaired insulin secretion (but not insulin contents) and decreased viability as measured by MTT but did not affect apoptosis or necrosis. Hence, decreased viability as assessed by MTT probably reflects functional mitochondrial impairment rather than loss of mitochondria. In line herewith the negative effects on glucose‐induced insulin secretion were reversible. More serious and irreversible effects could be envisaged after an extended exposure time to hyperoxia, something which was not tested here.

Komatsu et al. (2016) reported a positive effect by hyperoxia (tested up to 50% oxygen) on the viability of human islets. Using rat islets, we did not detect any positive effects of culture at 30 or 40% oxygen (unpublished observations). In another study, culture of human islets *below* atmospheric oxygen was reported to induce optimal functionality (Fraker et al. 2013). A discrepancy between studies could be due to differences in oxygen concentrations between studies as well as other factors; here we tested for effects after 24 h, whereas the published report (Komatsu et al. 2016) tested after 7 days of oxygen exposure. Also, base‐line viability status of islets may have differed. Hence, the loss of islets seemed higher during culture in the previous study (50%) than in ours.

When measuring proteins of mitochondrial complexes we detected, as previously in rat islets (Ma et al. 2014), signs of mitochondrial adaptability, which in human islets consisted in a lowering of mitochondrial complex II and an increase in complex III and IV. To further elucidate how hyperoxia interacts with beta cell mitochondria we turned to INS‐1 cells. These cells of rat origin resemble native beta cells in being responsive to glucose in terms of insulin secretion. Relevance of results in INS‐1 cells for the situation in human beta cells was further strengthened by the negative impact of hyperoxia in both types of cells (although glucose‐induced insulin was not clearly affected in the clonal cells) as well as partial similarities on effects on mitochondrial complexes in INS‐1 cells and human islets.

Using INS‐1 cells for measurements of oxygen consumption, we found in intact cells that 24 h of hyperoxia led to a decrease in basal oxygen consumption. Moreover, residual oxygen consumption following oligomycin was increased (relative to basal consumption), indicating enhanced mitochondrial uncoupling. We then tested whether such or similar effects could be evoked by the acute introduction of hyperoxia to cells. Remarkably, we found, to our knowledge for the first time, that the presence of hyperoxia exerted an almost immediate and dramatic effect on mitochondrial function; an effect that resembled effects recorded following a 24 h exposure to hyperoxia. Hence, ambient hyperoxia reduced basal respiration without any obvious time delay; this was followed by a probable uncoupling effect on oxygen consumption as assessed after the introduction of oligomycin 20–30 min later. Also, the results from permeabilized cells are compatible with an uncoupling effect. In any case, our findings highlight a moment‐to‐moment flexibility of mitochondrial function that is probably vital for normal functioning of all types of cells. Successful flexibility may underlie our findings that ATP contents of INS‐1 cells were not affected by hyperoxia; however, decreased demands for the ATP‐consuming processes of insulin secretion and biosynthesis may also be important.

Intuitively, the swift uncoupling effect that we observe during, as well as after hyperoxia should be beneficial in counteracting harmful deviations from the normal oxidative phosphorylation series of events. Interestingly, we previously found the opposite effect, i.e. a decreased degree of uncoupling after hypoxia, thereby enhancing the efficiency of oxidative phosphorylation under conditions of scarcity of oxygen. Also, whereas hyperoxia downregulated complex I (in INS‐1 cells) and complex II (in human islets) opposite effects were found in rat and human islets and in INS‐1 cells following hypoxia (Hals et al. 2015). We speculate that similar mechanisms are governing increased uncoupling during hyperoxia and decreased uncoupling during hypoxia. This notion remains to be investigated as well as the precise molecular mechanisms underlying the swift changes in uncoupling that we observe.

The experiments in neonatal mice strongly indicate that negative effects of hyperoxia on beta cells are operative not only in vitro but also in vivo. Thus in islets from hyperoxia‐exposed pups we find a significant reduction in insulin secretion of islets in the first 12–18 h following isolation of islets. These effects appear to be purely functional and reversible since we could not detect any abnormalities in insulin secretion in vitro when tested for later during normoxia. Also, glucose tolerance testing at age 10 weeks, that is, 8 weeks after hyperoxia exposure did not reveal any abnormality. However, these findings do not completely rule out an influence on beta cells later during adulthood.

Altogether our results in vitro and in vivo observations give support for the notion that negative effects of hyperoxia on beta cells may be operative during the treatment of extremely preterm babies. It is well recognized that hyperglycemia frequently occurs in extremely preterm babies (Hey 2005). To the best of our knowledge, the possibility that hyperoxia could be one factor behind the hyperglycemia has not been mentioned or evaluated in previous publications. A related question pertains to whether hyperoxia treatment during neonatal age could influence glucose metabolism and in particular beta cell function later in life. We had the opportunity to assess glucose metabolism in adults who, because of extreme prematurity, had been subjected to hyperoxia, usually for several days or weeks. Multiple factors are bound to influence glucose metabolism during postnatal life, however, for this study we still deemed it of interest to analyze parameters reflecting glucose homeostasis, insulin resistance and insulin sensitivity in a cohort of preterm born individuals, at age 25–28 years. These results demonstrate a higher level of HbA1c in these individuals. Although well within nondiabetic reference limits (none of the subjects had diabetes), the difference suggests a lesser efficiency in the regulation of glucose metabolism. This could be due at least in part to insulin resistance and resistance was indeed indicated by the calculated HOMA2‐IR parameter and the HOMA‐%S parameter. Our results showing insulin resistance are in agreement with a previous study (Hovi et al. 2007). Our HOMA results failed to show reduced beta cell function. However, the crudeness of HOMA parameters, in particular HOMA‐%B, has to be recognized. Studies encompassing insulin stimulatory tests would be necessary to assess beta cell function in our cohorts more rigorously.

In conclusion, hyperoxia in vitro exerts negative effects on human beta cells. Hyperoxia interacts swiftly with beta cell mitochondria leading to reduced oxygen consumption and increased uncoupling. Results in mouse neonates indicate that hyperoxia in vivo negatively affects beta cells in terms of insulin secretion. Taken together these findings open the possibility of negative effects on beta cells during hyperoxia treatment in preterm babies.

## Conflict of Interest

None declared.

## References

[phy213447-bib-0001] Balasuriya, C. , K. A. I. Evensen , M. P. Mosti , A. M. Brubakk , G. W. Jacobsen , M. S. Indredavik , et al. 2017 Peak bone mass and bone microarchitecture in adults born with low birth weight preterm or at term: a cohort study. J. Clin. Endocrinol. Metab. 102:2491–2500. 28453635 10.1210/jc.2016-3827

[phy213447-bib-0002] Bjorklund, A. , and V. Grill . 1993 B‐cell insensitivity in vitro: reversal by diazoxide entails more than one event in stimulus‐secretion coupling. Endocrinology 132:1319–1328. 7679978 10.1210/endo.132.3.7679978

[phy213447-bib-0003] Cantley, J. , S. T. Grey , P. H. Maxwell , and D. J. Withers . 2010 The hypoxia response pathway and beta‐cell function. Diabetes Obes. Metab. 12(Suppl 2):159–167. 21029313 10.1111/j.1463-1326.2010.01276.x

[phy213447-bib-0004] Dionne, K. E. , C. K. Colton , and M. L. Yarmush . 1993 Effect of hypoxia on insulin secretion by isolated rat and canine islets of Langerhans. Diabetes 42:12–21. 8420809 10.2337/diab.42.1.12

[phy213447-bib-0005] Evensen, K. A. , J. Skranes , A. M. Brubakk , and T. Vik . 2009 Predictive value of early motor evaluation in preterm very low birth weight and term small for gestational age children. Early Hum. Dev. 85:511–518. 19450939 10.1016/j.earlhumdev.2009.04.007

[phy213447-bib-0006] Fraker, C. A. , S. Cechin , S. Alvarez‐Cubela , F. Echeverri , A. Bernal , R. Poo , et al. 2013 A physiological pattern of oxygenation using perfluorocarbon‐based culture devices maximizes pancreatic islet viability and enhances beta‐cell function. Cell Transplant. 22:1723–1733. 23068091 10.3727/096368912X657873

[phy213447-bib-0007] Goto, M. , T. M. Eich , M. Felldin , A. Foss , R. Kallen , K. Salmela , et al. 2004 Refinement of the automated method for human islet isolation and presentation of a closed system for in vitro islet culture. Transplantation 78:1367–1375. 15548977 10.1097/01.tp.0000140882.53773.dc

[phy213447-bib-0008] Hals, I. K. , S. G. Bruerberg , Z. Ma , H. Scholz , A. Bjorklund , and V. Grill . 2015 Mitochondrial respiration in insulin‐producing beta‐cells: general characteristics and adaptive effects of hypoxia. PLoS ONE 10:e0138558. 26401848 10.1371/journal.pone.0138558 PMC4581632

[phy213447-bib-0009] Herbert, V. , K. S. Lau , C. W. Gottlieb , and S. J. Bleicher . 1965 Coated charcoal immunoassay of insulin. J. Clin. Endocrinol. Metab. 25:1375–1384. 5320561 10.1210/jcem-25-10-1375

[phy213447-bib-0010] Hey, E. 2005 Hyperglycaemia and the very preterm baby. Semin. Fetal Neonatal. Med. 10:377–387. 15927546 10.1016/j.siny.2005.04.008

[phy213447-bib-0011] Hohmeier, H. E. , H. Mulder , G. Chen , R. Henkel‐Rieger , M. Prentki , and C. B. Newgard . 2000 Isolation of INS‐1‐derived cell lines with robust ATP‐sensitive K+ channel‐dependent and ‐independent glucose‐stimulated insulin secretion. Diabetes 49:424–430. 10868964 10.2337/diabetes.49.3.424

[phy213447-bib-0012] Hovi, P. , S. Andersson , J. G. Eriksson , A. L. Jarvenpaa , S. Strang‐Karlsson , O. Makitie , et al. 2007 Glucose regulation in young adults with very low birth weight. N. Engl. J. Med. 356:2053–2063. 17507704 10.1056/NEJMoa067187

[phy213447-bib-0013] Komatsu, H. , D. Kang , L. Medrano , A. Barriga , D. Mendez , J. Rawson , et al. 2016 Isolated human islets require hyperoxia to maintain islet mass, metabolism, and function. Biochem. Biophys. Res. Commun. 470:534–538. 26801563 10.1016/j.bbrc.2016.01.110

[phy213447-bib-0014] Kulkarni, A. C. , P. Kuppusamy , and N. Parinandi . 2007 Oxygen, the lead actor in the pathophysiologic drama: enactment of the trinity of normoxia, hypoxia, and hyperoxia in disease and therapy. Antioxid. Redox Signal. 9:1717–1730. 17822371 10.1089/ars.2007.1724

[phy213447-bib-0015] Lohaugen, G. C. , A. Gramstad , K. A. Evensen , M. Martinussen , S. Lindqvist , M. Indredavik , et al. 2010 Cognitive profile in young adults born preterm at very low birthweight. Dev. Med. Child Neurol. 52:1133–1138. 21175467 10.1111/j.1469-8749.2010.03743.x

[phy213447-bib-0016] Ma, Z. , N. Moruzzi , S. B. Catrina , V. Grill , and A. Bjorklund . 2014 Hyperoxia inhibits glucose‐induced insulin secretion and mitochondrial metabolism in rat pancreatic islets. Biochem. Biophys. Res. Commun. 443:223–228. 24299957 10.1016/j.bbrc.2013.11.088

[phy213447-bib-0017] Mosmann, T. 1983 Rapid colorimetric assay for cellular growth and survival: application to proliferation and cytotoxicity assays. J. Immunol. Methods 65:55–63. 6606682 10.1016/0022-1759(83)90303-4

[phy213447-bib-0018] Ohki, T. , Y. Sato , T. Yoshizawa , K. Yamamura , K. Yamada , and K. Yamagata . 2012 Identification of hepatocyte growth factor activator (Hgfac) gene as a target of HNF1alpha in mouse beta‐cells. Biochem. Biophys. Res. Commun. 425:619–624. 22877752 10.1016/j.bbrc.2012.07.134

[phy213447-bib-0019] The Oxford Center for Diabetes . Endocrinology & Metabolism. Diabetes Trial Unit. HOMA Calculator. Available from https://www.dtu.ox.ac.uk/homacalculator/index.php. 2013.

